# INTERVENTIONS TO IMPROVE HEALTH AND QUALITY OF LIFE

**DOI:** 10.1093/geroni/igad104.1745

**Published:** 2023-12-21

## Abstract

Presenters in this session describe research related to efforts to promote health and well-being from biological, psychological, and social perspectives.

